# Uukuniemi Virus as a Tick-Borne Virus Model

**DOI:** 10.1128/JVI.00095-16

**Published:** 2016-07-11

**Authors:** Magalie Mazelier, Ronan Nicolas Rouxel, Michael Zumstein, Roberta Mancini, Lesley Bell-Sakyi, Pierre-Yves Lozach

**Affiliations:** a CellNetworks Cluster of Excellence and Department of Infectious Diseases, Virology, University Hospital Heidelberg, Heidelberg, Germany; b INRS–Institut Armand-Frappier, Université du Québec, Laval, Canada; c ETH Zurich, Institute of Biochemistry, Zurich, Switzerland; d The Pirbright Institute, Pirbright, United Kingdom; University of Pittsburgh School of Medicine

## Abstract

In the last decade, novel tick-borne pathogenic phleboviruses in the family Bunyaviridae, all closely related to Uukuniemi virus (UUKV), have emerged on different continents. To reproduce the tick-mammal switch *in vitro*, we first established a reverse genetics system to rescue UUKV with a genome close to that of the authentic virus isolated from the Ixodes ricinus tick reservoir. The IRE/CTVM19 and IRE/CTVM20 cell lines, both derived from I. ricinus, were susceptible to the virus rescued from plasmid DNAs and supported production of the virus over many weeks, indicating that infection was persistent. The glycoprotein G_C_ was mainly highly mannosylated on tick cell-derived viral progeny. The second envelope viral protein, G_N_, carried mostly *N*-glycans not recognized by the classical glycosidases peptide-*N*-glycosidase F (PNGase F) and endoglycosidase H (Endo H). Treatment with β-mercaptoethanol did not impact the apparent molecular weight of G_N_. On viruses originating from mammalian BHK-21 cells, G_N_ glycosylations were exclusively sensitive to PNGase F, and the electrophoretic mobility of the protein was substantially slower after the reduction of disulfide bonds. Furthermore, the amount of viral nucleoprotein per focus forming unit differed markedly whether viruses were produced in tick or BHK-21 cells, suggesting a higher infectivity for tick cell-derived viruses. Together, our results indicate that UUKV particles derived from vector tick cells have glycosylation and structural specificities that may influence the initial infection in mammalian hosts. This study also highlights the importance of working with viruses originating from arthropod vector cells in investigations of the cell biology of arbovirus transmission and entry into mammalian hosts.

**IMPORTANCE** Tick-borne phleboviruses represent a growing threat to humans globally. Although ticks are important vectors of infectious emerging diseases, previous studies have mainly involved virus stocks produced in mammalian cells. This limitation tends to minimize the importance of host alternation in virus transmission to humans and initial infection at the molecular level. With this study, we have developed an *in vitro* tick cell-based model that allows production of the tick-borne Uukuniemi virus to high titers. Using this system, we found that virions derived from tick cells have specific structural properties and *N*-glycans that may enhance virus infectivity for mammalian cells. By shedding light on molecular aspects of tick-derived viral particles, our data illustrate the importance of considering the host switch in studying early virus-mammalian receptor/cell interactions. The information gained here lays the basis for future research on not only tick-borne phleboviruses but also all viruses and other pathogens transmitted by ticks.

## INTRODUCTION

The Bunyaviridae are the largest family of RNA viruses. With over 350 members distributed worldwide and classified into five genera (Tospovirus, Nairovirus, Orthobunyavirus, Phlebovirus, and Hantavirus), these viruses represent a global threat to public health, livestock, and agricultural productivity (1). Hantaviruses apart, bunyaviruses are mainly transmitted by arthropods, including sandflies, mosquitoes, and ticks. In the past 5 years, a number of emerging tick-borne phleboviruses have been reported on different continents. Severe fever with thrombocytopenia syndrome virus (SFTSV) in Asia and Heartland virus (HRTV) in North America are recent examples of new tick-borne phleboviruses causing severe and often fatal disease in humans (2–5). Other phleboviruses genetically related to SFTSV and HRTV have also been recently isolated from ticks in different parts of the world (6). The increasing number of outbreaks and the apparent wide distribution in tick reservoirs demonstrate that these viruses must be taken seriously as emerging agents of disease. Currently, no vaccines or treatments are approved for human use.

Tick-borne phleboviruses are enveloped, roughly spherical viruses, with a diameter of about 100 nm, and have a tripartite, single-stranded RNA genome that exclusively replicates in the cytosol (7). The large (L) RNA segment codes for the viral RNA-dependent RNA polymerase L, and the medium (M) segment codes for the glycoproteins G_N_ and G_C_, both in a negative-sense orientation in the viral genomic RNA (vRNA) (7, 8). In addition to the vector, one of the main distinctions between tick-borne phleboviruses and the phleboviruses vectored by dipterans or mosquitoes is the absence of a sequence encoding a nonstructural protein in the M segment, NSm (7, 8). The small RNA segment (S) codes for the nucleoprotein N and the nonstructural protein NSs in an ambisense manner (7, 8). The proteins N and NSs are translated from subgenomic mRNAs transcribed from the vRNA and the antigenomic, replicative-intermediate RNA (cRNA), respectively (7, 8). In the virions, the protein N is associated with the virus RNA genome and, together with the polymerase L, constitutes the ribonucleoproteins (RNPs) (7). In the virus envelope, the glycoproteins G_N_ and G_C_ form spike-like projections responsible for virus attachment to host cells and for acid-activated penetration by membrane fusion from late endosomal compartments (7, 9).

During natural transmission to vertebrates, tick-borne phleboviruses are introduced into the skin dermis of hosts following bites by infected ticks. Although ticks are both harmful parasites and vectors of several emerging diseases, not only those caused by phleboviruses, little information on the cell biology of ticks is available (10–14). As a consequence, the composition and structure of tick cell-derived phleboviral particles remain largely undefined. Our knowledge of the initial infection of vertebrate hosts, cellular receptors, and cell entry is mainly based on the use of virus stocks produced in mammalian cells. SFTSV has been shown to subvert the nonmuscle myosin heavy chain IIA for early steps of infection (15). Rhabdoviral particles pseudotyped with the glycoproteins of SFTSV (SFTSV-pp) have been found to infect macrophages (16). Human dendritic cells (DCs) are productively infected by many bunyaviruses, including Uukuniemi virus (UUKV), a phlebovirus originally isolated from the tick Ixodes ricinus in the 1960s (17–21). To enter and infect DCs, SFTSV-pp and UUKV have been shown to exploit DC-SIGN, a C-type (calcium-dependent) lectin that binds high-mannose *N*-glycans on the viral glycoproteins through its C-terminal carbohydrate recognition domain (CRD) (16, 17). Interactions with L-SIGN, a C-type lectin closely related to DC-SIGN but expressed in liver sinusoidal endothelial cells, have also been recently documented for SFTSV-pp and UUKV (16, 22). After binding, SFTSV-pp and UUKV depend on endosomal acidification for penetration and infection (16, 23). UUKV is a late-penetrating virus, belonging to a large group of viruses that depend on late endosomal maturation for productive entry (9, 23).

We focus here on UUKV, a virus that shares high sequence homology with SFTSV and HRTV (8, 24, 25). However, UUKV is not associated with any disease in humans and is a validated biosafety level 2 (BSL2) surrogate for arthropod-borne bunyaviruses of higher biosafety classification (26). Major advances into various aspects of the phlebovirus life cycle, including virion structure, receptors, cell entry, and assembly, have been achieved through the use of UUKV (9, 23, 27–32). To determine whether tick cells support phlebovirus productive infection *in vitro* and whether tick cell-derived phleboviral particles infect human or other mammalian cells, we rescued UUKV from cDNAs with a genome close to that of the authentic virus isolated from ticks. Using this system, we examined infection and virus production in tick cells, assessed the progeny virus for interactions with DC-SIGN expressed in mammalian cells, analyzed the glycans and the electrophoretic mobility of the virus glycoproteins G_N_ and G_C_, and tested the infectivity of viral progeny in mammalian cells.

## MATERIALS AND METHODS

### Cells and viruses.

All products used for cell culture were obtained from Life Technologies or Sigma-Aldrich. Baby hamster kidney cells (BHK-21) were grown in Glasgow's minimal essential medium (GMEM) supplemented with 10% tryptose phosphate broth, 5% fetal bovine serum (FBS), 1% GlutaMAX, 100 units · ml^−1^ penicillin, and 100 μg · ml^−1^ streptomycin (33). Human B (Raji) and epithelial (HeLa) cells that stably express DC-SIGN were cultured according to ATCC recommendations (17, 34). All mammalian cell lines were grown in an atmosphere of 5% CO_2_ in air at 37°C. The tick cell lines IRE/CTVM19 and IRE/CTVM20 were cultured in L-15-based medium in sealed, flat-sided tubes (Nunc) in ambient air at 28°C as reported elsewhere (35–37). The prototype UUKV strain 23 (UUKV S23) was originally isolated from the tick I. ricinus in the 1960s (i.e., the virus in tick suspension) (21). The UUKV strain used in this study results from five successive plaque purifications of UUKV S23 in chicken embryo fibroblasts (CEFs) and subsequent passages in BHK-21 cells (38, 39). Virus multiplicity of infection is given according to the titer determined in BHK-21 cells.

### Antibodies and reagents.

The mouse monoclonal antibodies 8B11A3, 6G9E5, and 3D8B3 are directed against the UUKV nucleoprotein N and the glycoproteins G_N_ and G_C_, respectively (40). The rabbit polyclonal antibodies K1224 and K5 are directed against the UUKV glycoproteins G_N_ and G_C_, respectively (41). All of these antibodies were a kind gift from Anna Överby and the Ludwig Institute for Cancer Research (Stockholm, Sweden). The rabbit polyclonal antibody U2 has been described previously and recognizes the UUKV proteins N, G_N_, and G_C_ (17). The neutralizing anti-DC-SIGN mouse monoclonal antibody IgG2a (mAb1621) was purchased from R&D Systems. NH_4_Cl and EDTA were obtained from Sigma-Aldrich and dissolved in deionized water.

### Plasmids.

The expression plasmids pUUK-N and pUUK-L were a kind gift from Anna Överby and code for, respectively, the UUKV nucleoprotein N and polymerase L (39). The cDNAs corresponding to the S, M, and L segments of UUKV were synthesized by reverse transcription-PCR (RT-PCR) from vRNA extracts of purified virus stock using the reverse transcriptase Superscript III (Life Technologies). Their amplification as a single PCR product was carried out using Herculase II fusion DNA polymerase (Agilent). The PCR products were then cloned between the murine polymerase I (Pol I) RNA polymerase promoter and terminator sequences in the pRF108 vector (a generous gift from Ramon Flick, Bioprotection Systems Corporation) (30). The resulting Pol I-driven plasmids (pRF108-S, pRF108-M, and pRF108-L) encoded each of the antigenomic UUKV RNA molecules (i.e., S, M, and L segments). The point mutation G2386A in the M segment was obtained with a QuikChange XL site-directed mutagenesis kit (Agilent) using the plasmid pRF108-M as a template. The complete list of primers and restriction enzymes used for cloning and mutagenesis is shown in Table 1.

**TABLE 1:** Names and sequences of the primers used for cloning and mutagenesis

Primer	Sense^*a*^	Sequence (5′ → 3′)^*b*^	Purpose^*c*^
RT-S	Forw.	*ACACAAAGACCTCCAACTTAGCTATCG*	RT, S segment
RT-M	Forw.	*ACACAAAGACGGCTAACATGGTAAGG*	RT, M segment
RT-L	Forw.	*ACACAAAGACGCCAAGATGCTTTTAGCG*	RT, L segment
UUKV-S-5NC	Forw.	AATCGTCTCTAGGT*ACACAAAGACCTCCAACTTAGCTATCG*	Cloning the S segment into pRF108 (pRF108-S)
UUKV-S-3NC	Rev.	AATCGTCTCTGGG*ACACAAAGACCCTCC*	
UUKV-M-5NC	Forw.	AATCGTCTCTAGGT*ACACAAAGACGGCTAACATGGTAAGG*	Cloning the M segment into pRF108 (pRF108-M)
UUKV-M-3NC	Rev.	AATCGTCTCGGGG*ACACAAAGACACGGCTACATGG*	
UUKV-L-5NC	Forw.	AATCGTCTCTAGGT*ACACAAAGACGCCAAGATGCTTTTAGCG*	Cloning the L segment into pRF108 (pRF108-L)
UUKV-L-3NC	Rev.	AATCGTCTCGGGG*ACACAAAGTCCGCCAAGATGGAAGTAAAGG*	
Mut-M-S	Forw.	*CAAGGATTCAGTGGATTGTC***A***ATCATCAATCATAGATCCCA*	Mutagenesis of the M segment (G2386A)
Mut-M-AS	Rev.	*TGGGATCTATGATTGATGAT***T***GACAATCCACTGAATCCTTG*	

a Forw, forward; Rev, reverse.

b The virus RNA sequence that is targeted is in italics, and the sequences introduced for cloning are in roman type. Underlined nucleotide sequences indicate a BsmBI restriction site. Bold nucleotides are the point mutations introduced in the M segment sequence of the UUKV lab strain.

c RT, reverse transcription.

### Rescue of UUKV from plasmid DNAs.

UUKV was rescued by transfecting BHK-21 cells (0.6 × 10^6^) with the expression plasmids pUUK-L (1 μg) and pUUK-N (0.3 μg) together with 0.5 μg each of pRF108-S, pRF108-M, and pRF108-L. Transfection was performed in the presence of Lipofectamine 2000 (Life Technologies) using a ratio of 3.8 μl to 1 μg of plasmids in 400 μl of Opti-MEM (Life Technologies). At 1 h posttransfection, complete GMEM containing 2% FBS was added to the cells. After 5 days, supernatants were collected, clarified, and titrated as described below. Rescued viruses were passaged a minimum of five times in BHK-21 cells.

### Virus titration by focus-forming assay.

Following infection of confluent monolayers with 10-fold dilutions of virus in FBS-free medium, cells were grown in the presence of medium containing 5% serum and supplemented with 0.8% carboxymethyl-cellulose to prevent virus spread. Foci were revealed with a diaminobenzidine solution kit (Vector Laboratories) after two-step immunostaining with the antibody U2 (1:1,000) and an anti-rabbit horseradish peroxidase-conjugated secondary antibody (Vector Laboratories) (23).

### Deglycosylation.

To assess the glycosylation pattern of the UUKV glycoproteins, virus stocks purified through a 25% sucrose cushion were denatured and exposed to one of the five following treatments: 1,000 units of endoglycosidase H (Endo H; Promega), 5 units of peptide-*N*-glycosidase F (PNGase F), 0.005 units of α-2(3, 6, 8, 9)-neuraminidase, 0.003 units of β-1,4-galactosidase, and 0.05 units of β-*N*-acetylglucosaminidase (all enzymes from Merck Millipore) according to the manufacturer's recommendations. Samples were then analyzed by SDS-PAGE on a 4 to 12% or 10% Bis-Tris NuPAGE Novex gel (Life Technologies) and immunoblotting.

### Protein analysis.

Viral protein extracts from virus stocks purified through a 25% sucrose cushion were analyzed by SDS-PAGE (Nu-PAGE Novex 4 to 12% Bis-Tris gel; Life Technologies) and transferred to a polyvinylidene difluoride (PVDF) membrane (iBlot transfer stacks; Life Technologies) (17, 23). When indicated in the text, purified viruses were pretreated with glycosidases or β-mercaptoethanol (40%). For Western blot analysis, the PVDF membranes were first incubated with primary mouse monoclonal antibody 8B11A3 (1:2,000), 3D8B3 (1:100), or 6G9E5 (1:100) or with rabbit polyclonal antibody K5 (1:250 to 1,000), 1224 (1:500 to 1,000) or U2 (1:2,000), all diluted in Tris-buffered saline (TBS) containing 0.1% Tween and 5% milk, and then with an anti-mouse or anti-rabbit horseradish peroxidase-conjugated secondary antibody (1:10,000; Santa Cruz), respectively. Bound antibodies were detected by exposure to enhanced chemiluminescence reagents (ECL; GE Healthcare or Life Technologies). For quantitative detection of viral proteins, membranes were first incubated with the rabbit polyclonal antibody U2 (1:1,000) and then with an anti-rabbit infrared fluorescence (IRDye) secondary antibody and analyzed with an Odyssey imaging system and the software Image Studio (Li-Cor Biosciences).

### Infection assays.

Mammalian cells were infected with virus at different multiplicities of infection (MOIs) in medium without FBS at 37°C for 1 h. Virus supernatant was then replaced by complete culture medium, and cultures were incubated for up to 64 h before fixation. Tick cells were exposed to viruses at different MOIs in culture medium containing FBS at 28°C for up to 48 h. When used for microscopy, tick cells were seeded on poly-l-lysine (0.01%)-coated coverslips at 28°C on the day before infection. For inhibition assays, cells were pretreated with inhibitors at different concentrations for 30 min and exposed to UUKV in the continuous presence of the inhibitors. The infection was monitored by either wide-field fluorescence microscopy or flow cytometry.

### Flow cytometry.

The flow cytometry-based infection assay has been described previously (23). Briefly, after fixation and permeabilization with 0.1% saponin, infected cells were incubated with the mouse monoclonal antibody 8B11A3 (1:400), 6G9E5 (1:400), or 3D8B3 (1:500) at room temperature for 1 h, washed, and subsequently exposed to Alexa Fluor 647 (AF647)-conjugated secondary anti-mouse antibodies (1:500; Life Technologies) at room temperature for 1 h. When the mouse monoclonal antibody 1621 (25 μg · ml^−1^) was used in infection assays to neutralize DC-SIGN, UUKV-infected cells were immunostained with the rabbit polyclonal antibody U2 (1:400) and AF647-conjugated secondary anti-rabbit antibody (1:500; Life Technologies). Flow cytometry-based analysis involved the use of a FACSCalibur cytometer (Becton Dickinson) and FlowJo software (TreeStar).

### Fluorescence microscopy.

Infected cells were fixed and permeabilized with phosphate-buffered saline (PBS) containing 0.1% Triton X-100 (Merck Millipore), incubated with the mouse monoclonal antibody 8B11A3 (1:1,000) at room temperature for 1 h, washed, and then exposed to AF488-conjugated secondary anti-mouse (1:800; Life Technologies) at room temperature for 1 h. Nuclei were subsequently stained with Hoechst 33258 (0.5 μg · ml^−1^; Life Technologies). Infection was quantified by counting cells in three independent fields, and cells were imaged with an Olympus IX81 microscope.

### Statistical analysis.

The data shown are representative of at least three independent experiments. Values are given as the means of triplicates ± standard deviations (SD).

### Sequencing of the full-length M segment isolated from UUKV-infected ticks.

Questing nymphs of the tick I. ricinus were collected in the region of Ramsvik and Hindens Rev (Sweden; 2013). Pools of 25 nymphs were homogenized, and the total RNA was extracted with a magnetic bead-based protocol as described elsewhere (kind gift of Janne Chirico, National Veterinary Institute, Uppsala, Sweden, and Sara Moutailler, ANSES, Maisons-Alfort, France) (42). The cDNA corresponding to the M segment of UUKV was synthesized by RT-PCR with the reverse transcriptase Superscript III (Life Technologies) and the specific primer RT-M (Table 1) before amplification as a single PCR product using the *Pfu* DNA polymerase (Promega) and the primers UUKV-M-5NC and UUKV-M-3NC (Table 1). PCR products were analyzed with a capillary sequencer by ABI (Eurofins Scientific).

### Nucleotide sequence accession numbers.

The GenBank accession numbers for the nucleotide sequences of the M segments of the tick isolates RVS and HRS are KX219593 and KX219594, respectively.

## RESULTS

### Recovery of UUKV S23 from RNA Pol I-driven plasmid DNAs.

**FIG 1:**
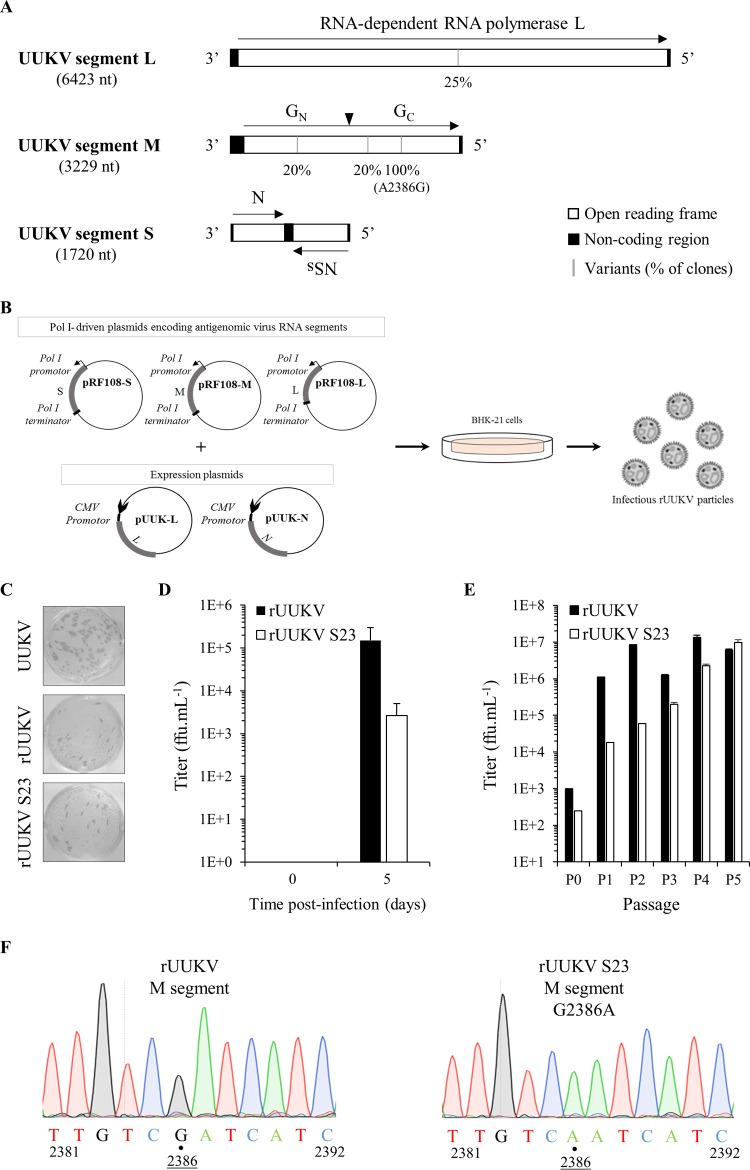
Recovery of UUKV from Pol I-driven plasmid DNAs. (A) The trisegmented, negative-sense RNA genome of UUKV. The black arrowhead shows the cleavage site in the polyprotein precursor of the glycoproteins G_N_ and G_C_. The black bars indicate the nucleotides found to be mutated in the UUKV laboratory strain relative to the sequence of UUKV strain 23. The penetrance of each mutation is indicated underneath. Four to five clones were analyzed for each viral genome segment. (B) Schematic representation of the Pol I-driven UUKV rescue system. (C) Focus-forming assay used for the titration of UUKV strains. Examples are shown for the UUKV lab strain (UUKV) and the viruses rescued from plasmid DNAs after five passages in BHK-21 cells (rUUKV and rUUKV S23). After 3 days of incubation at 37°C, foci were immunostained with the rabbit polyclonal antibody U2 against the viral proteins N, G_N_, and G_C_. (D) rUUKV and rUUKV S23 production 5 days after transfection of plasmids expressing L, M, and S segments under the control of the Pol I promoter together with the UUKV L and N expression plasmids pUUK-L and pUUK-N in BHK-21 cells. (E) Titer of rUUKV and rUUKV S23 after rescue (passage 0, P0) and up to five passages (P1 to P5) in BHK-21 cells. FFU, focus-forming units. (F) Sequence analysis of the rUUKV S23 M segment compared to that of rUUKV carried out from vRNA purified extracts after five passages in BHK-21 cells.

The UUKV lab strain that we used in this study as a template for cloning purposes results from the adaptation of the prototype tick isolate strain 23 (UUKV S23) to BHK-21 cells after successive plaque purifications in CEFs (21, 38, 39). Compared with the S, M, and L nucleotide sequences published for the original UUKV S23 that was plaque purified five times in CEFs (GenBank accession numbers NC_005221.1, NC_005220.1, and NC_005214.1, respectively), we identified only a few mutations in our UUKV lab strain (Fig. 1A). Most were in the M segment. Over the entire virus genome, only one mutation was conserved, a nonsilent substitution (A2386G) in the M transcript (43–45) (Fig. 1A). In all further experiments, for convenience, UUKV will refer to our current mammalian cell culture-adapted lab strain, rUUKV will refer to the same virus but rescued from cDNAs, UUKV S23 will refer to the original tick isolate plaque purified five times in CEFs, and rUUKV S23 will refer to the viral particles produced from plasmids encoding genome sequences identical to those published for UUKV S23 in the late 1980s and early 1990s (43–45).

Using a reverse genetics system that relies on the cellular Pol I promoter for the synthesis of viral transcripts, the anti-genomic full-length segments S, M, and L from UUKV were cloned into the vector pRF108, which contains the Pol I promoter and terminator. This system has been successfully employed to synthesize chimeric transcripts of the UUKV segment M (30) and to recover infectious particles of the mosquito-borne phlebovirus Rift Valley fever virus (RVFV) from plasmid DNAs (46, 47). The complete system is depicted in Fig. 1B. From the plasmids coding for the genome of UUKV, it was possible to obtain all the segments encoding UUKV S23 with only one site-directed mutagenesis (G2386A in the M segment). This reversion results in the addition of an arginine instead of a glutamine at position 276 in the sequence of the glycoprotein G_C_.

As there is no evidence for Pol I promoter activity in tick cells, both rUUKV and rUUKV S23 were first rescued from BHK-21 cells, which are highly permissive to most bunyaviruses. The infectious progenies were detected in the cell culture medium by a focus-forming assay (Fig. 1C). Both focus formation and growth properties of the recombinant rUUKV and rUUKV S23 in BHK-21 cells were similar to those of UUKV (Fig. 1C). Cotransfection of the Pol I-driven full-length S, M, and L plasmids together with plasmids coding for the viral polymerase L and nucleoprotein N, whose expression depends on the cytomegalovirus promoter, was essential for the recovery of infectious particles (data not shown).

The level of infectious rUUKV and rUUKV S23 in the cell supernatant remained relatively modest 5 days after transfection, from thousands to hundreds of thousands focus-forming units (FFU) per milliliter (Fig. 1D). However, the titers significantly increased over subsequent rounds of amplification in BHK-21 cells and reached a plateau of 10^7^ FFU · ml^−1^ after 2 to 3 passages, typical for UUKV (17, 23) (Fig. 1E). To assess the identity of the recombinant virus strains recovered from cDNAs, we used the point reversion G2386A in the M sequence as a genetic marker. Total vRNA from rUUKV or rUUKV S23 was extracted after 5 passages in BHK-21 cells, reverse transcribed, and sequenced with primers spanning the reversion site (Fig. 1F). The sequencing showed that rUUKV S23 was derived from the transfected plasmids and not from contaminating UUKV.

**FIG 2:**
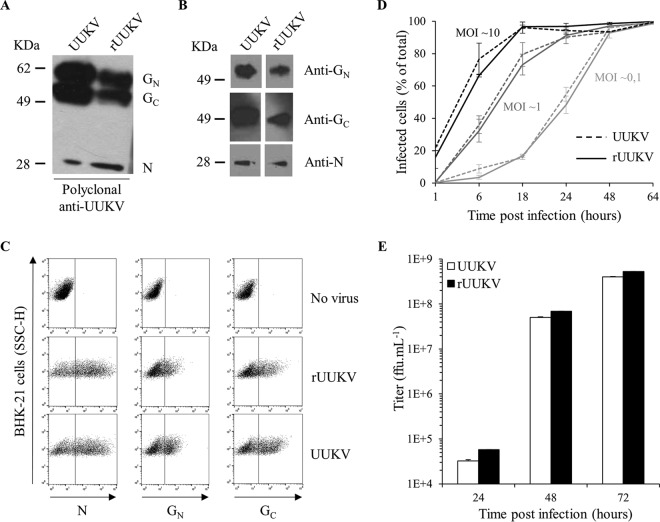
Characterization of UUKV rescued from plasmids. The UUKV lab strain and rUUKV were analyzed by SDS-PAGE and Western blotting under reducing conditions (A) using the rabbit polyclonal antibody U2 against the three structural viral proteins N, G_N_, and G_C_ or under nonreducing conditions (B) with the mouse monoclonal antibodies 8B11A3, 6G9E5, and 3D8B3 that recognize each of the structural proteins N, G_N_, and G_C_, respectively. (C) BHK-21 cells were exposed to the UUKV lab strain or rUUKV at an MOI of 0.1 for 24 h. After fixation and permeabilization, infected cells were immunostained for N, G_N_, and G_C_ with the mouse monoclonal antibodies 8B11A3, 6G9E5, and 3D8B3, respectively, and analyzed by flow cytometry. SSC-H, side scatter, height. (D) Infection of BHK-21 cells by UUKV and rUUKV was monitored over 64 h using the flow cytometry-based assay used for the experiment shown in panel C. Infection is given as the percentage of N protein-positive cells. (E) Supernatants collected from cells infected at an MOI of 0.1 and at indicated times were assessed for the production of infectious viral progeny by focus-forming assay.

We next analyzed rUUKV particles, infection, replication, and progeny production in BHK-21 cells in comparison to those of UUKV. When the viruses were subjected to SDS-PAGE and Western blotting, all three major structural proteins, namely, N, G_N_, and G_C_, were observed in both rUUKV and UUKV virions (Fig. 2A and B). To monitor infection, we used mouse monoclonal antibodies against each of the newly synthesized virus proteins, nucleoprotein N and glycoproteins G_N_ and G_C_, before flow cytometry analysis (Fig. 2C). Using N protein expression as the readout, we found that the kinetics of rUUKV infection were quite similar to those of infection with UUKV (Fig. 2D). The increase in the proportion of infected cells over time emphasized that viral replication, and not input virus, was quantified in these assays. A complete cycle, from binding to release of infectious progeny, lasted about 6 to 7 h in BHK-21 cells (data not shown) and reached a plateau after 48 h (Fig. 2E). Similar results were obtained with rUUKV S23 (data not shown).

Taken together, our results show that infectious viruses with genomic RNAs identical to those of the original UUKV S23 can be recovered from cDNAs. In turn, our reverse genetics system can be confidently used to investigate the life cycle of tick-borne phleboviruses in vector tick cells as well as the subsequent transmission and initial infection in mammalian hosts. Because the recombinant rUUKV S23 is the model closest to the authentic tick isolate, we then focused on this strain.

### Tick cells are persistently infected by UUKV.

**FIG 3:**
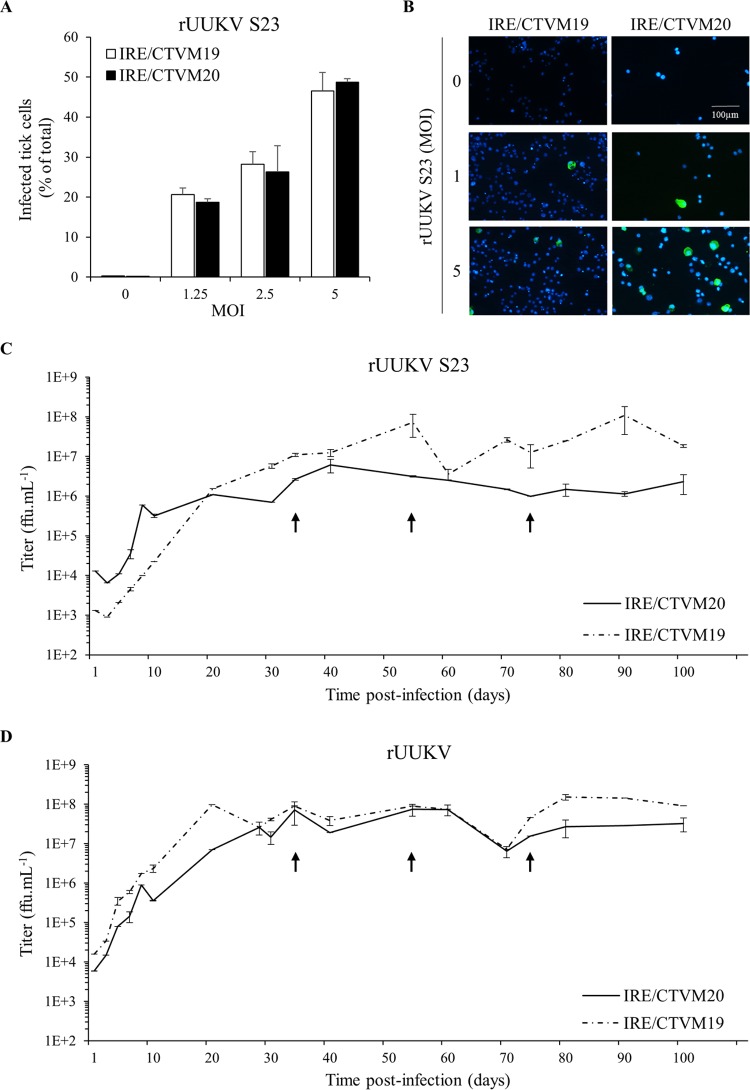
Infection of tick cells by rUUKV and rUUKV S23 is persistent. (A) Tick cell lines IRE/CTVM19 and IRE/CTVM20 were exposed to BHK-21 cell-derived rUUKV S23 at the indicated MOIs for 48 h. Infection was analyzed by flow cytometry after immunostaining against the nucleoprotein N. (B) IRE/CTVM19 and IRE/CTVM20 cells were exposed to various MOIs of rUUKV S23 derived from BHK-21 cells. The next day, infected cells were immunostained for the intracellular UUKV nucleoprotein N using the anti-N primary mouse monoclonal antibody 8B11A3 and an AF488-coupled anti-mouse secondary monoclonal antibody (green). Nuclei were stained with Hoechst (blue), and samples were analyzed by wide-field microscopy. (C and D) IRE/CTVM19 and IRE/CTVM20 cells were exposed to rUUKV S23 and rUUKV, as indicated. Supernatant (200 μl) of infected cells was harvested daily during the first 10 days and every 10 days thereafter. The production of infectious viral particles in the supernatant was determined by focus-forming assays. The cells were subcultured in fresh complete medium (1:1) after sampling of the parent cells on days 34, 54, and 74 (black arrows).

rUUKV S23 rescued in BHK-21 cells was used to infect tick cells and regenerate virions with all the features of the original tick-derived virus (e.g., lipids, glycosylation, etc.). Two cell lines derived from embryos of I. ricinus, the tick species from which UUKV was first isolated, were used: IRE/CTVM19 and IRE/CTVM20 (35), obtained from the Tick Cell Biobank at The Pirbright Institute, United Kingdom. To determine whether IRE/CTVM19 and IRE/CTVM20 cells support infection by tick-borne phleboviruses, they were exposed to rUUKV S23 for 48 h and immunostained with mouse monoclonal antibodies against the nucleoprotein N of UUKV. Flow cytometry allowed quantitative detection of infected cells. Independent of the tick cell line, nearly 50% of cells were infected at a multiplicity of infection (MOI) of 5, and about 20% were infected at an MOI as low as 1.25 (Fig. 3A). The sensitivity of tick cells to rUUKV S23 infection was confirmed by fluorescence microscopy after immunostaining of the newly synthesized virus nucleoprotein N (Fig. 3B). These experiments showed that both tick cell lines support infection of rUUKV S23.

We next assessed infected cells for the production of virus progeny. Although cells were infected, titration of cell-free supernatants collected from challenged tick cells indicated that the production of infectious rUUKV S23 particles was almost nonexistent within the first 24 h of infection (data not shown). After this period, the amount of infectious progeny produced from both tick cell lines increased over time to reach a plateau value within 3 weeks (Fig. 3C). Subculturing of infected tick cells 34, 54, and 74 days postinfection, consisting of the removal of half of the cell suspension and replacement with fresh culture medium, did not result in a decrease in viral progeny production (indicated by black arrows in Fig. 3C). At this time, no cytopathic effects were observed following subculture, and the cells grew normally. At 100 days postinfection, high levels of infectious virus were still detectable despite the subculturing of cells. Similar results were obtained when rUUKV was used to infect IRE/CTVM19 and IRE/CTVM20 cells (Fig. 3D). After several months of amplification in tick cells, the reversion G/A introduced at position 2386 in the M segment was still present in the genome of rUUKV S23 but not in that of rUUKV (data not shown). Altogether these data indicate that I. ricinus cells support persistent infection by recombinant UUKV.

### Mammalian cells support infection by rUUKV S23 grown in tick cells.

**FIG 4:**
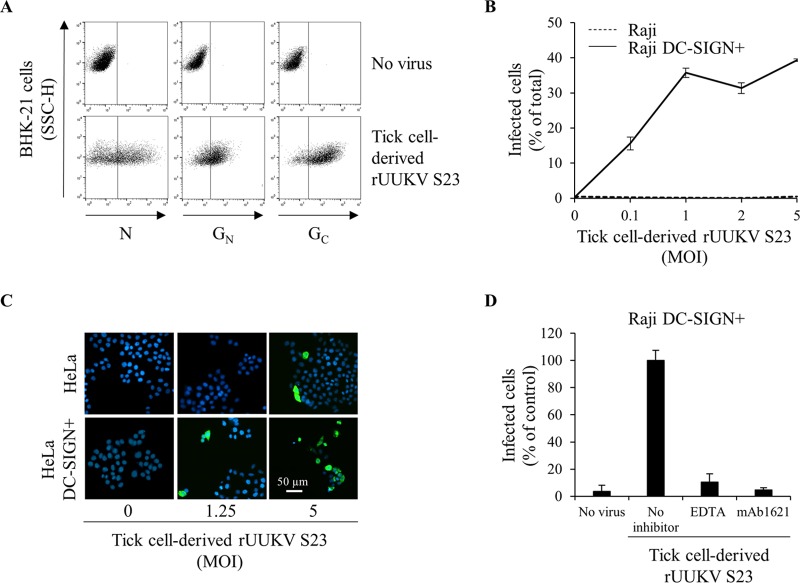
The C type lectin DC-SIGN enhances infection of human cells by tick cell-derived rUUKV S23. (A) BHK-21 cells were infected (at an MOI of 0.1) with rUUKV S23 derived from IRE/CTVM19 cells for 18 h and immunostained for N, G_N_, and G_C_ proteins prior to flow cytometry analysis. (B) Parental (Raji) and DC-SIGN-expressing Raji cells (Raji DC-SIGN+) were infected with IRE/CTVM19 cell-derived rUUKV S23 and analyzed by flow cytometry 16 h after immunostaining against the viral nucleoprotein. (C) Parental (HeLa) and DC-SIGN-expressing HeLa cells (HeLa DC-SIGN+) were exposed to various MOIs of IRE/CTVM19 cell-derived rUUKV S23. The next day, infected cells were immunostained for the intracellular virus nucleoprotein N using the anti-N primary mouse monoclonal antibody 8B11A3 and an AF488-coupled anti-mouse secondary monoclonal antibody (green). Nuclei were stained with Hoechst (blue), and samples were analyzed by wide-field microscopy. (D) Raji DC-SIGN-expressing cells were exposed to IRE/CTVM19 cell-derived rUUKV S23 (MOI of ∼1) in the presence of inhibitors blocking DC-SIGN, namely, EDTA (5 mM) or the neutralizing mouse monoclonal antibody mAb1621 (25 μg · ml^−1^). Intracellular viral antigens were detected by immunostaining with an anti-UUKV rabbit polyclonal antibody, followed by incubation with AF647-conjugated secondary antibodies. Infection was analyzed by flow cytometry 18 h later and normalized to infection of DC-SIGN-expressing Raji cells in the absence of inhibitor (as a percentage of the control).

To examine whether rUUKV S23 derived from tick cells remains able to infect mammalian cells, BHK-21 cells were exposed to the viral progeny derived from IRE/CTVM19 cells for 18 h. Infected cells were analyzed with conformation-dependent mouse monoclonal antibodies in a flow cytometry-based assay. A large proportion of the infected cells were positive for both the virus nucleoprotein N and glycoproteins G_N_ and G_C_ (Fig. 4A), suggesting that infection leads to viral replication. Therefore, our phlebovirus-tick cell system appears to be an excellent model for reproducing transmission of tick-borne viruses to mammalian hosts *in vitro*.

### DC-SIGN mediates infection by tick cell-derived rUUKV S23.

Due to the presence of dermal DCs at the site of initial infection via tick bite, they are among the first cells to encounter the incoming viruses (1, 48). We recently established that DC-SIGN, which is highly expressed on the surface of human dermal DCs, binds UUKV directly via interactions with high-mannose *N*-glycans on the virus glycoproteins (17). The capacity of DC-SIGN to bind tick cell-derived viral particles was therefore evaluated using Raji and HeLa cells stably expressing the lectin after transduction with a TRIPΔU3 lentiviral vector encoding human DC-SIGN (34). Raji and HeLa cells normally have low or no sensitivity to phlebovirus infection. As expected, parental Raji cells were not detectably infected with IRE/CTVM19 cell-derived rUUKV S23 at an MOI of 5 or less (Fig. 4B). However, when DC-SIGN was expressed, up to 40% of Raji cells became infected at an MOI of 0.1 (Fig. 4B). Similarly, fluorescence microscopy analysis of HeLa cells exposed to various MOIs of the virus showed that infection was greatly increased when the lectin was expressed (Fig. 4C).

To confirm that the infection was mediated by DC-SIGN, we utilized the neutralizing mouse monoclonal antibody mAb1621 and EDTA, which inhibits the DC-SIGN binding function by extracting the bound calcium (33). The increase in infectivity due to DC-SIGN expression was significantly reduced in cells treated with inhibitors (Fig. 4D). Together, these results clearly indicate that infection by tick cell-derived rUUKV S23 is mediated by DC-SIGN and suggest that the viral glycoproteins have, at least in part, high-mannose carbohydrates recognized by the lectin.

### G_N_ and G_C_ present distinct glycosylation profiles on tick and mammalian cell-derived rUUKV S23 particles.

**FIG 5:**
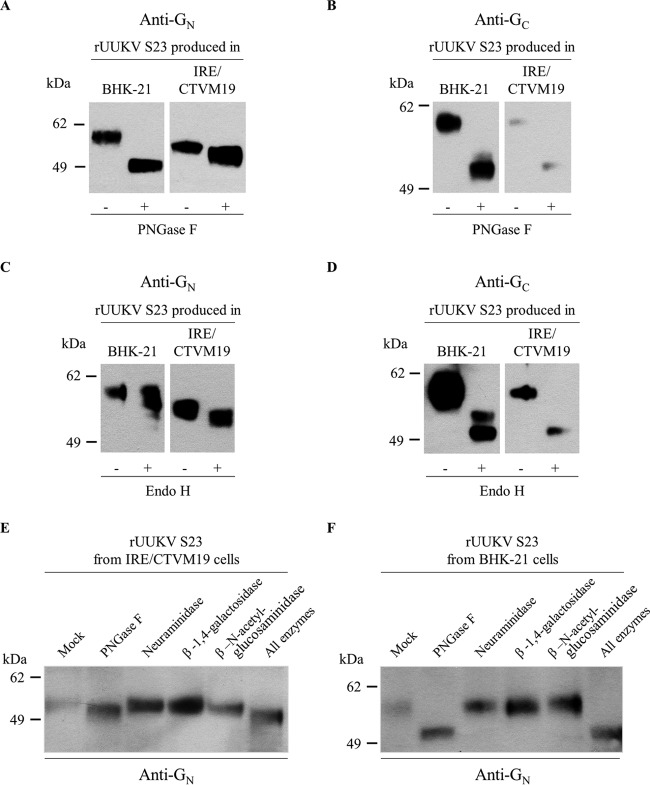
Glycosylation of the rUUKV S23 envelope glycoproteins G_N_ and G_C_ on virions produced from tick and mammalian cells. IRE/CTVM19 and BHK-21 cell-derived rUUKV S23 purified through a 25% sucrose cushion was reduced and denatured before digestion with PNGase F (A, B, E, and F), Endo H (C and D), or neuraminidase, β-1,4-galactosidase, and β-*N*-acetylglucosaminidase (E and F). Proteins were analyzed by SDS-PAGE and Western blotting using the rabbit polyclonal antibodies K1224 and K5 against linear epitopes in the viral glycoproteins G_N_ (A, C, E, and F) and G_C_ (B and D), respectively.

Like other bunyaviruses, UUKV has several *N*-linked oligosaccharides in its envelope glycoproteins G_N_ and G_C_ (four sites each). To analyze the glycosylation pattern of G_N_ and G_C_ on tick cell-derived viruses, rUUKV S23 was treated with peptide-*N*-glycosidase F (PNGase F) under denaturing conditions before SDS-PAGE and Western blotting. When rUUKV S23 was produced in IRE/CTVM19 cells, at least one *N*-glycosylation site on G_N_ appeared sensitive to PNGase F, while all four sites were sensitive when the virus was derived from BHK-21 cells (Fig. 5A). In contrast, all four of the *N*-glycans on the glycoprotein G_C_ were susceptible to the glycosidase regardless of the cells used to produce rUUKV S23 (Fig. 5B).

To further examine the *N*-glycans on rUUKV S23, the virus was subjected to endoglycosidase H (Endo H). When rUUKV S23 was amplified in BHK-21 cells, the glycoprotein G_N_ acquired complex glycosylation and was mainly Endo H resistant (Fig. 5C), while G_C_ carried mainly high-mannose *N*-glycans, as evidenced by sensitivity to Endo H digestion (Fig. 5D); i.e., only one site remained resistant. When the virus was produced in the IRE/CTVM19 tick line, both glycoproteins G_N_ and G_C_ were sensitive to Endo H, with a digestion pattern identical to that of PNGase F (Fig. 5C and D). These results indicate that all the *N*-glycans on G_N_ and G_C_ on tick cell-derived virus particles contain, at least, high-mannose core structures.

Of the two viral envelope glycoproteins, G_N_ shows more striking differences in glycosylation patterns between tick and BHK-21 cells. To further address these distinctions, rUUKV S23 was exposed to neuraminidase, β-1,4-galactosidase, and β-*N*-acetylglucosaminidase, which liberate neuraminic acids, β-galactosides, and terminal β-*N*-acetylglucosamine and *N*-acetylgalactosamine residues from oligosaccharides, respectively. G_N_ originating from tick cells, but not from BHK-21 cells, was sensitive only to β-*N*-acetylglucosaminidase to a similar extent as PNGase F (Fig. 5E and F). Overall, our data indicate that UUKV particles derived from vector tick cells have glycosylation profiles distinct from those in mammalian cells.

### The reduction of disulfide bonds impacts the electrophoresis mobility of G_N_ derived from mammalian cells but not from tick cells.

**FIG 6:**
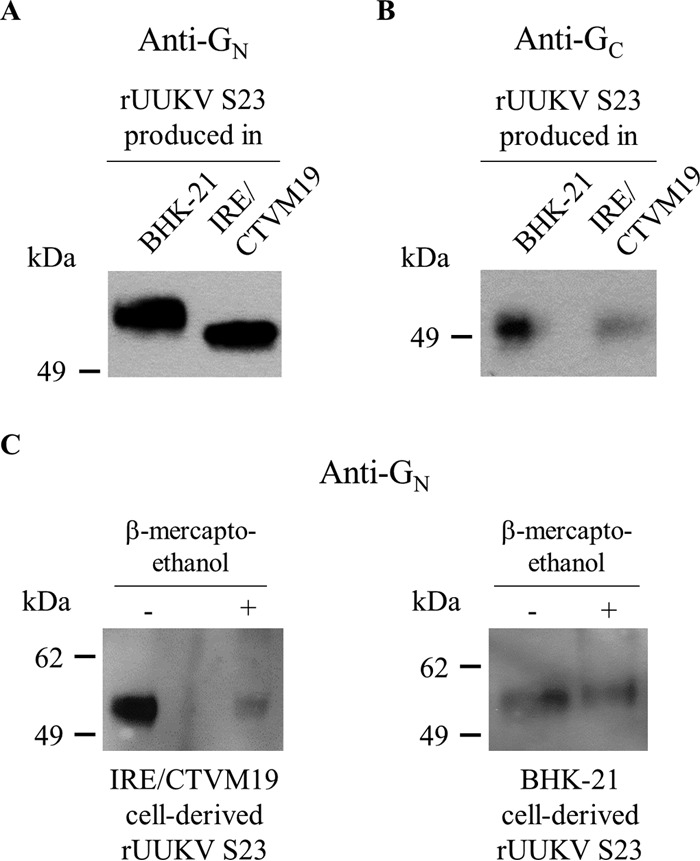
Electrophoretic mobility of the glycoproteins G_N_ and G_C_ on rUUKV S23 produced in tick and mammalian cells. (A and B) BHK-21 and IRE/CTVM19 cell-derived rUUKV S23 purified through a 25% sucrose cushion were analyzed by nonreducing SDS-PAGE and Western blotting, using the mouse monoclonal antibodies 6G9E5 and 3D8B3 against conformational epitopes in G_N_ (A) and G_C_ (B), respectively. (C) rUUKV S23 viruses produced in BHK-21 and IRE/CTVM19 cells were purified through a 25% sucrose cushion and analyzed by nonreducing (−) or reducing (+) SDS-PAGE and Western blotting with the rabbit polyclonal anti-G_N_ antibody K1224.

Under reducing and nonreducing conditions, the electrophoretic mobility of G_N_ on tick cell-derived rUUKV S23 appeared faster than that of the protein on viral particles originating from BHK-21 cells (Fig. 5A and 6A, respectively). In contrast, the electrophoretic mobility of G_C_ on tick cell-derived virions appeared similar to that of the glycoprotein on viruses produced from BHK-21 cells, under either reducing or nonreducing conditions (Fig. 5B and 6B, respectively).

We next assessed whether treatment with β-mercaptoethanol results in a change in the apparent molecular weight of G_N_ made in tick and mammalian cells. When virions produced in BHK-21 cells were analyzed, the electrophoretic mobility of G_N_ appeared slower under reducing conditions (Fig. 6C). Under our experimental conditions, no shift was observed when the protein originated from particles derived from tick cells, suggesting that the number of disulfide bonds differs between G_N_ on tick and mammalian cell-derived viruses. Altogether these results suggest that the viral glycoprotein G_N_ on viruses produced in tick cells has different maturation and folding properties than those of the protein on viral particles derived from mammalian cells.

### The structural proteins G_N_, G_C_, and N in infectious viral progeny produced in tick and mammalian cells.

**FIG 7:**
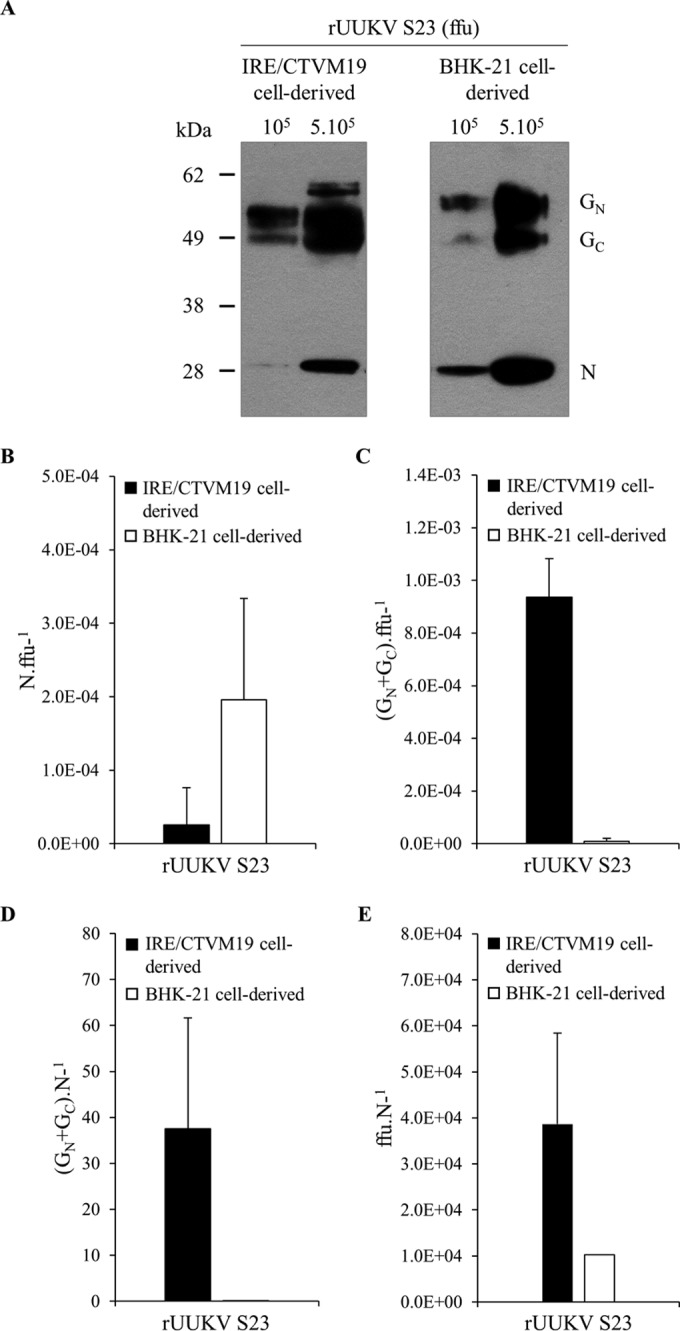
The structural rUUKV S23 proteins G_N_, G_C_, and N in infectious particles derived from tick and mammalian cells. (A) The amounts of viral glycoproteins and N protein for 10^5^ and 5 × 10^5^ focus forming units (FFU) of purified rUUKV S23, produced in either tick cells or BHK-21 cells, were analyzed by SDS-PAGE and Western blotting using the rabbit polyclonal anti-UUKV antibody U2, which recognized G_N_, G_C_, and N. (B to E) The amount of viral glycoproteins and protein N for identical amounts of purified infectious rUUKV S23 was determined by quantitative Western blotting (Odyssey Imaging Systems) with the anti-UUKV U2 and an anti-rabbit infrared fluorescence secondary antibody. The ratios of the amounts of N protein (B) or viral glycoproteins (C) per FFU and the ratios of the amounts of viral glycoproteins (D) or number of FFU (E) per relative unit of N protein are shown.

We then further examined potential differences in the structure of rUUKV S23 particles derived from tick and mammalian cells. For this purpose, viruses were purified through a 25% sucrose cushion to retain only viral particles with an intact envelope. Identical numbers of infectious particles, based on titration on BHK-21 cells, were analyzed by SDS-PAGE and Western blotting using the polyclonal antibody U2 against the whole virus, thereby enabling the detection of G_N_, G_C_, and N. The amounts of nucleoprotein N and glycoproteins G_N_ and G_C_ appeared markedly different in virions produced in tick and BHK-21 cells, with substantially less protein N and more glycoprotein G_N_ and G_C_ in tick cell-derived viruses (Fig. 7A).

The amounts of G_N_, G_C_, and N proteins incorporated into rUUKV S23 particles were determined by quantitative Western blotting using the anti-UUKV U2 and an anti-rabbit infrared fluorescence secondary antibody prior to analysis with an Odyssey imaging systems. The ratio between the amount of protein N and the number of FFU was significantly lower in tick cell-derived viruses (Fig. 7B) while that between the amount of viral glycoproteins and number of FFU was greater (Fig. 7C). It was also apparent that the numbers of G_N_ and G_C_ molecules per nucleoprotein N (Fig. 7D) and the number of FFU per N (Fig. 7E) were higher in virions originating from tick cells. Similar results were obtained with rUUKV (data not shown). From these results, it is most likely that the global structural organizations of viral particles exhibit differences between viruses produced in tick and mammalian cells.

### Wild-type UUKV originating from Swedish ticks in the 2010s.

All RNA viruses are known to have high polymerase error rates. To determine whether the wild-type UUKV in vector tick populations has genetically evolved since the 1960s and, thus, whether our tick-borne virus model is still representative of the circulating virus, we analyzed 16 pools of 25 nymphs of the tick I. ricinus recently collected in the region of Ramsvik and Hindens Rev (Sweden; 2013) for the presence of the UUKV RNA segment M. The cDNA corresponding to the full-length M segment of UUKV was first synthesized by RT-PCR from vRNA extracted with magnetic beads from nymphal homogenates and then amplified by a single PCR. Out of the 16 homogenates, 4 were positive, from which it was possible to obtain the full-length nucleotide M sequence for two samples (GenBank accession numbers KX219593 and KX219594). Due to limited amounts of material, a partial sequence was obtained only from one of the two other samples. Nucleotide sequence analysis showed identity greater than 93% between the UUKV S23 and the virus circulating currently. At the amino acid level, the sequence identity reached a minimum of 98.4%, with 7 to 11 amino acid variations located in G_N_ and 2 to 4 variations in G_C_ (Table 2). Together, our data indicate a modest genetic evolution of UUKV glycoproteins in ticks.

**TABLE 2:** Full-length sequence of the viral glycoproteins G_N_ and G_C_ obtained from UUKV-infected field Ixodes ricinus nymphal ticks

Region (aa)^*a*^	Isolate	Identity (%)^*b*^	Positive sequence (%)^*c*^	Substitution(s) relative to the UUKV S23 polypeptide precursor^*d*^
G_N_ (18–496)	RVS	98.5	99.6	**L8I**, **L29I**, **S124T**, **T167S**, **L207V**, A219T, T237A
	HRS	97.6	98.7	**L29I**, T44A, **S124T**, **T167S**, **L207V**, A219T, **S282T**, T287A, N476K, N477Q, A479C
Intraregion	RVS	100.0	100.0	
(497–513)	HRS	97.1	97.1	A508T
G_C_ (514–1008)	RVS	99.6	100.0	**S695T**, **Q790R**
	HRS	99.2	99.8	**K577R**, **Q790R**, **T841S**, L1003F

a Amino acid (aa) position.

b Percent identity relative to the sequence of the precursor peptide.

c Values represent the percent identity of the sequence of interest augmented by the substitutions shown in boldface, which are assumed to have a weak impact, relative to the sequence of the precursor protein.

d Total RNAs were extracted from 16 pools of 25 nymphs collected in the region of Ramsvik and Hindens Rev, Sweden, in 2013. The cDNA corresponding to the M segment of UUKV was synthesized by RT-PCR and amplified by a single PCR. Out of the 16 nymphal pools, four were positive for the virus, and from these four samples, one partial and two full-length nucleotide sequences could be obtained and. The list is shown of the amino acids found to be mutated in the full-length amino acid sequences of isolates RVS and HRS (GenBank accession numbers KX219593 and KX219594, respectively) relative to the sequence of the polypeptide precursor of the two glycoproteins G_N_ and G_C_ of UUKV S23 (GenBank accession number NC_005220.1). The point mutations in boldface indicate positive substitutions.

### Dependence on low pH of rUUKV S23 infectious entry.

**FIG 8:**
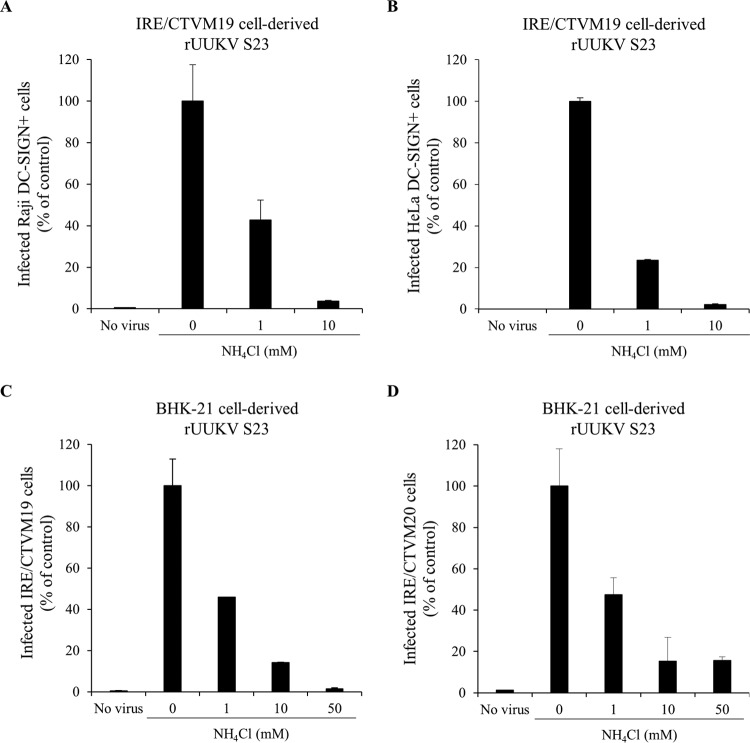
Low-pH dependence of rUUKV S23 for infection. (A and B) Raji and HeLa cells that stably express DC-SIGN were pretreated with NH_4_Cl, a weak base that neutralizes the endosomal pH, and then exposed to IRE/CTVM19 cell-derived rUUKV S23 (MOI of ∼5) in the continuous presence of the inhibitor. Infected cells were harvested 16 h later and immunostained for the UUKV nucleoprotein N. Infection was analyzed by flow cytometry (A) or wide-field microscopy counting at least 200 cells in more than three independent fields (B). Data were normalized to DC-SIGN-expressing cells infected in the absence of inhibitor (as a percentage of the control). (C and D) IRE/CTVM19 and IRE/CTVM20 cells were infected with BHK-21 cell-derived rUUKV S23 at an MOI of 5 for 36 h in the continuous presence of NH_4_Cl. Infection was analyzed by flow cytometry and normalized against data obtained in the absence of inhibitor (as a percentage of the control).

We have recently showed in human, rodent, and monkey cells that infectious entry of UUKV is pH dependent (23). We examined whether the envelope proteins G_N_ and G_C_ remain dependent on endosomal acidification to trigger the infectious entry of tick cell-derived viruses into mammalian cells. To this end, DC-SIGN-expressing Raji and HeLa cells were exposed to IRE/CTVM19 cell-derived rUUKV S23 in the presence of agents that neutralize vacuolar pH. The lysosomotropic weak base ammonium chloride (NH_4_Cl) induced a dose-dependent inhibition of infection in both cell lines (Fig. 8A and B). Infection of both IRE/CTVM19 and IRE/CTVM20 cells was also sensitive to NH_4_Cl (Fig. 8C and D). Similar results were obtained when chloroquine, another lysosomotropic agent, was employed (data not shown). These data show that rUUKV S23 infection relies on vacuolar acidification regardless of the host cell origin of the virus and the targeted cell type.

## DISCUSSION

Ticks belong to the Arachnida, a class distinct from that of insects (Insecta) (49, 50). They are arthropods of huge economic significance worldwide, both as harmful ectoparasites and as vectors of many agents of disease, including protozoan and helminth parasites, bacteria, and viruses (10, 50). As obligate parasites, tick-borne viruses share dependence on biochemical and biophysical features gained in ticks for production in arthropod vectors and then transmission to mammalian hosts and other vertebrates. However, very little is known about the molecular and cell biology of ticks. In the Bunyaviridae family, a number of novel pathogenic tick-borne phleboviruses, all closely related to UUKV, have recently emerged in different parts of the world (24, 25). In this context, there is renewed interest in using UUKV as a model system to examine the initial infection of human hosts by these emerging pathogenic viruses.

The orthobunyavirus Bunyamwera virus was the first bunyavirus for which a reverse genetics system was developed to allow the recovery of infectious viral particles from plasmid DNAs (51). Since then, similar or derivative methods have been adapted to some other bunyaviruses, including the tick-borne phlebovirus SFTSV (1, 52–55). In this study, we have added UUKV to the list with a reverse genetics system that relies on the use of the cellular RNA Pol I promoter, enabling modification of the viral genome. The UUKV particles recovered from plasmids were infectious and had replication characteristics similar to those of the authentic virus. An alternative approach to rescuing UUKV from cDNAs that utilizes the T7 promoter was recently described (56). In contrast to that method, our Pol I-driven system requires the cotransfection of plasmids coding for the proteins N and L to trigger viral replication and production. This may be explained by the fact that the RNA transcripts under the control of the T7 promoter are exclusively processed in the cytosol, while those regulated by the Pol I promoter follow the classical cellular mRNA maturation cycle through the nucleus. The main critical advantage of our system concerns the stability of the rescued virus, which we showed to be stable after long-term passage.

The reference UUKV strain 23 was isolated from the tick I. ricinus in the 1960s and then adapted to tissue culture, being passaged and amplified over many years, first in chicken embryo cells and subsequently in BHK-21 cells (23, 39, 57). However, the nucleotide sequences of the three RNA segments of our UUKV lab strain did not exhibit major evolution compared to those published for the initial virus. As a consequence of this conservation, remarkably high for an RNA virus, it was possible to obtain all the original UUKV RNA transcripts with only one site-directed mutagenesis in the sequence coding for the glycoprotein G_C_. In addition, this single mutation does not seem to confer any significant advantages to the virus in terms of infectivity and replication capacity in either tick or mammalian cells.

When UUKV S23 was compared with viruses currently circulating in tick populations, we found a rather modest number of point mutations in the nucleotide sequence of the M segment, which codes for the glycoproteins G_N_ and G_C_. Furthermore, most of these mutations were silent at the amino acid level, with sequence identities reaching 98% and higher. It is not possible to conclude whether the few differences result from (i) the genetic evolution of the virus since the 1960s, (ii) different sites for the collection of ticks (i.e., Finland for strain 23 and Sweden for the new isolates), or (iii) adaptation of strain 23 to tissue culture. However, while cloning the circulating virus in our reverse genetics system for future investigations into the new point mutations remains paramount, it is apparent that our tick cell-virus model already allows for addressing many aspects of both the cell biology of ticks and the early steps of initial infection in mammalian hosts, from virus transmission to entry.

Several bunyaviruses are vectored by ticks: a few orthobunyaviruses, all the nairoviruses, and all of the phleboviruses related to UUKV (1, 49, 58). While some tick cell lines have been shown to be sensitive to bunyaviruses, including the nairovirus Crimean-Congo hemorrhagic fever virus, we have extended these observations to tick-borne phleboviruses (49). We found that both the IRE/CTVM19 and IRE/CTVM20 cell lines originating from the tick I. ricinus are sensitive to UUKV. Both lines supported a complete virus life cycle, from infectious entry to release of new infectious progeny. Although the interaction of additional phleboviruses with tick cell lines must be assessed experimentally, it is likely that many lines can be infected by tick-borne phleboviruses.

Arthropod-borne viruses infecting humans and other animals are generally maintained in arthropod vectors and amplified in nonhuman vertebrates. This is also the case for bunyaviruses (1). In contrast to vertebrate hosts, there is no clear evidence of pathology or lethal outcomes following bunyavirus infection in arthropod hosts. In line with these observations, IRE/CTVM19 and IRE/CTVM20 cells infected by UUKV grew normally without any sign of cytopathic effects for many weeks, as reported previously for other arboviruses propagated in tick cell lines (49). Cells exposed to UUKV remained infected for months, demonstrating the asymptomatic persistence of UUKV infection in tick cells. This contrasts with the inability of mammalian cells to survive infection after a couple of days. In UUKV, we have found a suitable surrogate to study important emerging tick-borne pathogens such as SFTSV and HRTV in both tick vectors and mammalian host cells, as well as to reproduce host alternation *in vitro*.

Previous studies have mainly involved virus stocks produced in mammalian cells. As a consequence, tick-arbovirus interactions are poorly understood at the cellular level, and the characteristics of viral particles produced from ticks are largely unknown. Using our model system to produce UUKV particles with a genome and biophysical properties very close to those of the virus isolated from ticks, we applied immunoblotting-based approaches to analyze the envelope glycoproteins G_N_ and G_C_ on tick cell-derived progeny virions. To different extents, both UUKV glycoproteins were sensitive to PNGase F treatment. This highlights the presence of classical *N*-glycan structures on tick cell-derived viral glycoproteins, at least in part.

After synthesis in rodent cells, all of the eight *N*-glycosylation sites in the UUKV envelope proteins G_N_ and G_C_ were found to carry oligosaccharides, mainly high-mannose glycans, in previous studies (17, 23, 29). In the present study, DC-SIGN enhanced infection of human cells by viruses isolated from tick cells, indicating the existence of high-mannose *N*-glycans on tick cell-derived glycoproteins. The sensitivity of G_N_ and G_C_ to Endo H confirmed this presence. It is reasonable to assume that tick cell-derived viruses have the capacity to target cells expressing such lectinic virus receptors in the skin dermis following introduction into humans and other vertebrates by infected ticks and before subsequent spread throughout the host.

The glycoprotein G_N_ was much less sensitive to PNGase F and Endo H when the virus was isolated from IRE/CTVM19 cells than when it was isolated from BHK-21 cells. In addition, the β-*N*-acetylglucosaminidase recognized G_N_ glycosylations only on tick cell-derived virions. Though we cannot completely exclude differential glycosylation processes in tick cells, our data rather support the view that tick cell-derived G_N_ carries atypical glycans not recognized by the classical glycosidases PNGase F and Endo H. Very little is known about the nature of oligosaccharides on tick-derived glycoproteins (14, 59). Specific tick glycans may impact the initial infection of human hosts in the skin dermis by broadening the spectrum of potential receptors and target cells or by influencing subsequent entry pathways, especially those mediated by lectin receptors. For instance, DC-SIGN is able to trigger selective signal transduction pathways, which depend on the nature and glycosylation pattern of the captured antigens (60, 61). UUKV can use distinct lectin receptors for entering cells through different mechanisms (22). With regard to this, elucidating the type of glycans synthesized in tick cells and on derivative viruses remains of paramount importance.

Both glycoproteins G_N_ and G_C_ on virions originating from BHK-21 cells showed molecular features distinct from those on viruses produced in tick cells. G_C_ differed by one *N*-glycan site, which remained insensitive to Endo H in BHK-21 cells. The glycoprotein G_N_ had a higher degree of dissimilarity, with striking differences in glycans but also in electrophoretic mobility under reducing and nonreducing conditions. The apparent molecular weight of G_N_ was lower for viruses produced in IRE/CTVM19 cells than for those in BHK-21 cells. No deletion in the virus genome segment M, which could explain the translation of a smaller-sized protein, was found after several weeks of virus propagation in tick cells (data not shown). The smaller size of glycan units or divergent transcription and maturation processes in tick cells may be responsible for the variation in molecular weight. In addition, that only BHK-21 cell-derived G_N_ molecules were sensitive to β-mercaptoethanol indicates a difference in the number of disulfide bonds between the proteins made in tick and mammalian cells. It is likely that the folding of G_N_ on virions differs depending on whether they are produced in tick or mammalian cells.

It is clear from our data that viral particles derived from IRE/CTVM19 and BHK-21 cells exhibit substantial differences in the amount of viral structural proteins, with less nucleoprotein N and more G_N_ and G_C_ glycoproteins on virions produced in tick cells. It is tempting to postulate that the level of N protein correlates with the quantity of RNPs and the number of particles and, therefore, that the ratio between the number of FFU and amount of N reflects the ratio between infectious and noninfectious particles. This ratio is known to be low for phleboviruses produced in mammalian cells (23). Though additional experiments are needed to confirm this model, production in tick cells arguably confers an advantage in terms of infectivity to the virus, i.e., with a higher ratio between infectious and noninfectious particles.

The ratio between the amount of nucleoprotein N and the amount of glycoproteins G_N_ and G_C_ for tick cell-derived viruses significantly differs from that for viral particles produced in mammalian cells. It is reasonable to believe that virions originating from tick cells present an overall structure different from that of the particles derived from mammalian cells. Electron microscopy pictures and tomography-based approaches have shown pleomorphic virions heterogeneous in size for UUKV produced in BHK-21 cells, with spike-like projections of 5 to 10 nm composed of the two glycoproteins G_N_ and G_C_ (27, 32). Structural analyses of UUKV and other tick-borne viruses obtained from arthropod vector cells are still lacking.

As with other bunyaviruses, tick-borne phleboviruses rely on vacuolar acidification for infectious entry into mammalian cells (1, 9, 16, 23). The fusion of the virus envelope with the cell membranes is triggered following the acidic activation of the viral glycoproteins. We found that the presence of NH_4_Cl resulted in blocking UUKV infection of DC-SIGN-expressing human cells by tick cell-derived viral particles and of both tick cell lines by viruses produced from BHK-21 cells. In other words, regardless of the host cells that are targeted or the cells from which the virus originates, UUKV depends on low pH for penetration and infection. Structural data support the view that the phlebovirus glycoprotein G_C_ is the fusion protein (class II) (62). The possible roles of G_N_ in the entry and fusion processes of UUKV and other tick-borne phleboviruses and also in host alternation remain to be uncovered.

The lipid composition of the viral envelope, adaptive mutations in the virus genome, and the nature of oligosaccharides in the virus glycoproteins on tick cell-derived viruses arguably influence the initial infection in humans and other vertebrates and subsequent spread throughout the host. Tick cell lines and UUKV provide an interesting, functional model to investigate not only tick-borne phleboviruses but also many other intracellular pathogens transmitted by ticks. The results gained here will open avenues in research on tick vectors, the detailed cell biology of which remains a challenge for future work.
